# Relationship between Tic disorders and 41 inflammatory factors in circulating blood: a two-sample Mendelian randomization study

**DOI:** 10.1016/j.clinsp.2025.100649

**Published:** 2025-07-10

**Authors:** Ciai Lai, Guolin Huang, Xi Chen, Xionghan Lian, Xin Li, Wei He, Guangliang Luo, Aiyuan Cai

**Affiliations:** a Shenzhen Hospital, Guangzhou University of Chinese Medicine, Shenzhen, PR China; b Guangzhou University of Chinese Medicine, Guangzhou, PR China; c Fujian University of Traditional Chinese Medicine, Fuzhou, PR China

**Keywords:** Mendelian randomization, Tic disorders, Inflammatory factors, IL-17, MIF, PDGF-BB

## Abstract

**Objective:**

To investigate the causal associations between 41 circulating inflammatory factors and Tic Disorders (TD) via the Mendelian Randomization (MR) approach.

**Methods:**

Single-Nucleotide Polymorphisms (SNPs) related to 41 circulating inflammatory factors were obtained from published Genome-Wide Association Studies (GWASs). The outcome event, TD, was sourced from the FinnGen Biobank database. MR was employed to explore the causal relationship between these inflammatory factors and TD. Causal inference was performed via Inverse Variance Weighted (IVW), MR-Egger, and Weighted Median (WM) methods. Heterogeneity was assessed by Cochran's *Q* statistic and the leave-one-out method. Horizontal pleiotropy was examined with MR-Egger regression and MR-PRESSO. SNPs with horizontal pleiotropy were removed via the PhenoScanner database to ensure result reliability.

**Results:**

MR analysis revealed significant causal associations between three circulating inflammatory factors and TD. Increased levels of Interleukin-17 (IL-17) and macrophage Migration Inhibitory Factor (MIF) were associated with an increased risk of TD (OR = 2.329, 95 % CI [1.069–5.078], p = 0.033; OR = 2.267, 95 % CI [1.097–4.686], p = 0.027), whereas increased levels of Platelet-Derived Growth Factor BB (PDGF-BB) were linked to a reduced incidence of TD (OR = 0.750, 95 % CI [0.387–1.453], p = 0.023). No causal relationships were found for other inflammatory factors. No heterogeneity or horizontal pleiotropy was detected during the study, and the MR statistical power (power > 80 %) confirmed the reliability of these three findings.

**Conclusion:**

MR analysis revealed causal links between IL-17, MIF, PDGF-BB and TD, suggesting important clinical implications for the development of targeted prevention and treatment strategies for TD.

## Highlights

• IL-17, MIF, and PDGF-BB are causally linked to tic disorders.
• IL-17 and MIF increase TD risk, while PDGF-BB is protective.
• Study results are robust with strong statistical power.

## Introduction

Tic Disorders (TDs) (International Classification of Diseases [ICD]-11 disease code 8A05.0) are neurodevelopmental disorders characterized by involuntary, repetitive, sudden, rapid, and nonrhythmic motor and/or vocal tics that typically onset in childhood.^1^ According to the Diagnostic and Statistical Manual of Mental Disorders (DSM-5) and the 11th Revision of the International Classification of Diseases (ICD-11), TD can be classified as mild, moderate, or severe. There are three main types of TD: transient TD, chronic TD, and Tourette syndrome (TS).^2^ TD is a lifelong condition, and currently, clinical treatment for mild TD consists mainly of medical education and psychological support, with a lack of effective drug intervention. Without intervention, nearly 50 % of children with TD progress, and up to 20 % continue to experience tic symptoms into adulthood or throughout their lives.^3^^,^^4^ In 5 % to 10 % of cases, tics in TD children not only worsen in adulthood but also develop severe TD. The clinical manifestations of TD are diverse, often accompanied by various comorbidities, and the etiology is complex, with unclear pathogenic mechanisms.^5^ Currently, the drugs used to treat TD are mainly psychotropic medications.^6^ Although they may temporarily alleviate symptoms, long-term clinical findings suggest that their efficacy is poor, especially with dopamine receptor blockers, which may lead to extrapyramidal reactions, excessive sedation, and adverse effects on memory and cognition.^7^ Overall, the current diagnostic and treatment options for TD are insufficient to meet global medical needs. Therefore, research on potential strategies for the prevention and management of TD is necessary.

Chronic inflammation is a key contributor to the development of common diseases, including cardiovascular disorders, cancers, and neurofunctional impairments. A Genome-Wide Association Study (GWAS) revealed downregulated expression of neuronal genes in subjects, coupled with upregulated expression of genes related to microglial function, suggesting that inflammation may be a significant environmental factor in the pathophysiology of neurodevelopmental disorders.^8^ Compared with 15 % of brain cells, microglia play pivotal roles in synaptic pruning, neuronal differentiation, and neural circuit formation. Research indicates that persistent chronic exposure to inflammatory factors can disrupt microglial function. Furthermore, in studies focused on neurodevelopmental disorder-related conditions, the activation of microglia has been closely linked to dysregulation of the release of immune-inflammatory cytokines such as IL-6, IL-8, and IL-10.^9^ Prospective research involving 200 children with Tic Disorders (TDs) or Obsessive-Compulsive Disorder (OCD) demonstrated that maternal autoimmune diseases and inflammatory states were more common in children who developed TD/OCD than in control individuals.^10^ While associations between inflammatory factors and TD have been investigated, most studies involve clinical observations that are subject to considerable confounding factors. Consequently, the causal relationship between cellular inflammatory cytokines and TD remains elusive.

With the increasing use of large-scale GWAS, Mendelian Randomization (MR) has been used for causal inference of different phenotypes.^11^ MR is a human genetic tool that uses the random allocation of gene variants during gamete formation and conception to make causal inferences.^12^ In MR, Single-Nucleotide Polymorphisms (SNPs) associated with the exposure event can be used as Instrumental Variables (IVs). Because IVs are unrelated to other confounding factors, MR can assess the causal relationship between previously observed exposure and outcome events while effectively avoiding confounding bias in traditional epidemiological studies.^13^ In this context, this study uses two-sample MR to assess the causal relationship between 41 inflammatory factors in circulating blood and TD to gain a deeper understanding of the impact of inflammatory factors on TD and explore new approaches for the prevention and treatment of TD.

## Materials and methods

### Design

**Fig. 1:**
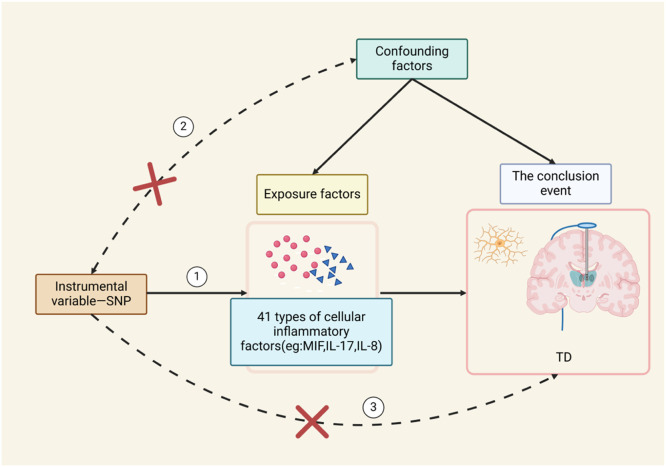
Schematic representation of Mendel's randomization hypothesis.

A two-sample Mendelian randomization study was conducted to infer causality, with 41 inflammatory factors in circulating blood as the exposure and TD as the outcome. The three main assumptions of MR are shown in Fig. 1. Assumption 1: the selected SNP is significantly associated with the exposure (41 inflammatory factors in circulating blood); Assumption 2: the SNP must be unrelated to potential confounders between the exposure and outcome; Assumption 3: the SNP is not directly related to the outcome TD and can only be causally associated through the 41 inflammatory factors in circulating blood.

### Exposure factors and outcome events GWAS data acquisition

The data used in this study are all from publicly available whole-genome association studies. Genetic analysis data for 41 inflammatory factors in circulating blood were obtained from the IEUOpenGWAS database (https://gwas.mrcieu.ac.uk/). The GWAS data for the outcome event TD were sourced from the FinnGenBiobank database (https://www.finngen.fi/en), published in 2021, with a total of 215,763 European ancestry samples, including 161 cases and 215,763 controls. For detailed information on the other data, please refer to Table 1.

**Table 1:** Data source information in Mendel's randomization study.

Exposure/Conclusion	Data Source	ID	The scope of ethnicity	Sample size	SNPs	Publication year
TD	FinnGen Biobank	finn-b-F5_TIC	European	215,763	16,380,457	2021
CCL27	Ieu open GWAS	ebi-a-GCST004420	European	3631	9568,408	2016
β-NGF	Ieu open GWAS	ebi-a-GCST004421	European	3531	9537,863	2016
VEGF	Ieu open GWAS	ebi-a-GCST004422	European	7118	9784,803	2016
MIF	Ieu open GWAS	ebi-a-GCST004423	European	3494	9537,573	2016
TRAIL	Ieu open GWAS	ebi-a-GCST004424	European	8186	9698,525	2016
TNF-β	Ieu open GWAS	ebi-a-GCST004425	European	1559	6304,298	2016
TNF-α	Ieu open GWAS	ebi-a-GCST004426	European	3454	9500,449	2016
CXCL12	Ieu open GWAS	ebi-a-GCST004427	European	5998	9736,366	2016
SCGF-β	Ieu open GWAS	ebi-a-GCST004428	European	3682	9574,890	2016
SCF	Ieu open GWAS	ebi-a-GCST004429	European	8290	9796,683	2016
IL-16	Ieu open GWAS	ebi-a-GCST004430	European	3483	9551,485	2016
RANTES	Ieu open GWAS	ebi-a-GCST004431	European	3421	9523,827	2016
PDGF-BB	Ieu open GWAS	ebi-a-GCST004432	European	8293	9800,009	2016
MIP-1β	Ieu open GWAS	ebi-a-GCST004433	European	8243	9802,973	2016
MIP-1α	Ieu open GWAS	ebi-a-GCST004434	European	3522	9519,267	2016
CXCL9	Ieu open GWAS	ebi-a-GCST004435	European	3685	9579,894	2016
M-CSF	Ieu open GWAS	ebi-a-GCST004436	European	840	9184,521	2016
MCP-3	Ieu open GWAS	ebi-a-GCST004437	European	843	7630,881	2016
MCP-1	Ieu open GWAS	ebi-a-GCST004438	European	8293	9801,908	2016
IL-12p70	Ieu open GWAS	ebi-a-GCST004439	European	8270	9799,886	2016
IP10	Ieu open GWAS	ebi-a-GCST004440	European	3685	9576,881	2016
IL-18	Ieu open GWAS	ebi-a-GCST004441	European	3636	9785,222	2016
IL-17	Ieu open GWAS	ebi-a-GCST004442	European	7760	9786,653	2016
IL-13	Ieu open GWAS	ebi-a-GCST004443	European	3557	9539,073	2016
IL-10	Ieu open GWAS	ebi-a-GCST004444	European	7681	9793,415	2016
IL-8	Ieu open GWAS	ebi-a-GCST004445	European	3526	9517,348	2016
IL-6	Ieu open GWAS	ebi-a-GCST004446	European	8189	9790,590	2016
IL-RA	Ieu open GWAS	ebi-a-GCST004447	European	3638	9564,741	2016
IL-1β	Ieu open GWAS	ebi-a-GCST004448	European	3309	9983,642	2016
HGF	Ieu open GWAS	ebi-a-GCST004449	European	8292	9802,538	2016
IL-9	Ieu open GWAS	ebi-a-GCST004450	European	3634	9567,876	2016
IL-7	Ieu open GWAS	ebi-a-GCST004451	European	3409	9692,306	2016
IL-5	Ieu open GWAS	ebi-a-GCST004452	European	3364	9450,731	2016
IL-4	Ieu open GWAS	ebi-a-GCST004453	European	8124	9786,064	2016
IL-2RA	Ieu open GWAS	ebi-a-GCST004454	European	3677	9583,519	2016
IL-2	Ieu open GWAS	ebi-a-GCST004455	European	3475	9512,914	2016
IFN-γ	Ieu open GWAS	ebi-a-GCST004456	European	7701	9785,363	2016
CXCL1	Ieu open GWAS	ebi-a-GCST004457	European	3505	9528,505	2016
CSF3	Ieu open GWAS	ebi-a-GCST004458	European	7904	9788,961	2016
bFGF	Ieu open GWAS	ebi-a-GCST004459	European	7565	9790,946	2016
CCL11	Ieu open GWAS	ebi-a-GCST004460	European	8153	9793,404	2016

## Methods

### Instrumental variables

In accordance with the STROBE-MR study guidelines,^19^ the following steps were taken to screen each SNP of the inflammatory factors: 1) Use a genome-wide significance threshold of p < 5×10^−8^, and if there were fewer significant SNPs under this standard, a threshold of p < 5×10^−6^ was used; 2) Conduct Linkage Disequilibrium (LD) testing via the clump function, with a standard set at r^2^ < 0.001, kb = 10,000; 3) Exclude SNPs related to the outcome via the PhenoScanner database (http://www.phenoscanner.medschl.cam.ac.uk/) to remove confounding factors; and 4) Calculate the *F* statistic for each SNP and exclude SNPs with *F* < 10 to avoid bias from weak instrumental variables. Additionally, the proportion of exposure explained by the instrumental variables (*R2*) was calculated to quantify the strength of the genetic instruments, with the following formula: R2=[2×Beta2×(1−EAF)×EAF]/[2×Beta2×(1−EAF)×EAF+2×SE2×N×(1−EAF)×EAF], where Beta represents the genetic effect of each SNP, EAF is the effect allele frequency, SE is the standard error, and *N* is the sample size. To assess the strength of the selected SNPs, the *F* statistic for each SNP was calculated via the following formula: F=R2(N−k−1)/k(1−R2), where *R2* represents the extent to which the selected SNP explains exposure, *N* represents the sample size, and k represents the number of instrumental variables included. Weak instrumental variables with *F* statistics less than 10 were removed. The remaining independent instrumental variables were used for subsequent MR analysis. 5) MR-PRESSO testing was conducted to detect outliers and adjust for horizontal pleiotropy. If horizontal pleiotropy was detected in the instrumental variables, outliers were removed.

### Two-sample Mendelian randomization analysis

Causal evaluations were conducted for each type of cell inflammatory factor associated with TD. The potential causal effects were assessed via the Inverse Variance-Weighted method (IVW), Weighted Median method (WM), and MR-Egger method and are presented as Odds Ratios (ORs) and 95 % Confidence Intervals (95 % CIs). To correct for multiple comparisons, a more stringent Bonferroni correction was applied with a threshold set at less than 0.05/n, where n represents the number of independent hypotheses. Heterogeneity was assessed via Cochran's *Q* test and leave-one-out method, with a nonsignificant Cochran's Q value (p > 0.05) indicating no heterogeneity and a significant value (p < 0.05) suggesting potential heterogeneity between genes. Horizontal pleiotropy was assessed via the MR-Egger method (with the intercept term) and the MR-PRESSO global test. The statistical analyses were primarily conducted via the TwoSampleMR package in R software (version 4.3.1). All reported P values were two-tailed, with p < 0.05 indicating significance. If there was no evidence of pleiotropy or heterogeneity, IVW was meaningful, as were the other methods, and the results were stable. The statistical power of MR (power > 80 %) was calculated via the mRnd tool on the website (https://shiny.cnsgenomics.com/).

## Results

### Instrumental variable selection results

SNPs that met the criteria of the three assumptions were selected, and variables that may affect the outcome were removed via the PhenoScanner database. The *F* values of the remaining instrumental variables were all greater than 10. When the genome-wide significance threshold of p < 5×10^−8^ was used as the standard, the number of usable SNPs was too small to analyze the results. Therefore, on the basis of the STROBE-MR study guidelines and literature review, the P value was set to p < 5×10^−6^. The IVW method was used to estimate the associations between 41 types of cytokines and TD. The analysis results revealed that elevated levels of Interleukin-17 (IL-17) and macrophage Migration Inhibitory Factor (MIF) may be associated with an increased risk of TD (OR = 2.329, 95 % CI [1.069–5.078], p = 0.033; OR = 2.267, 95 % CI [1.097–4.686], p = 0.027), whereas elevated levels of Platelet-Derived Growth Factor BB (PDGF-BB) were associated with a decreased incidence of TD (OR = 0.750, 95 % CI [0.387–1.453], p = 0.023). The specific results are shown in Table 2.

**Table 2:** MR analysis results of circulating blood cell inflammatory markers and the risk of TD.

	Numbers of SNPs	MR Egger			WM			IVW		
		SE	p	OR (95 % CI)	SE	p	OR (95 % CI)	SE	p	OR (95 % CI)
**IL-17**	9	0.767	0.147	3.481 (0.778‒15.582)	0.535	0.024	2.106 (0.738‒6.011)	0.082	0.033	2.329 (1.069‒5.078)
**MIF**	6	0.626	0.652	1.357 (0.397‒4.631)	0.462	0.025	2.280 (0.922‒5.641)	0.370	0.027	2.267 (1.097‒4.686)
**PDGF-BB**	4	0.897	0.036	0.687 (0.118‒3.985)	0.381	0.039	0.644 (0.305‒1.360)	0.337	0.023	0.750 (0.387‒1.453)

**Fig. 2:**
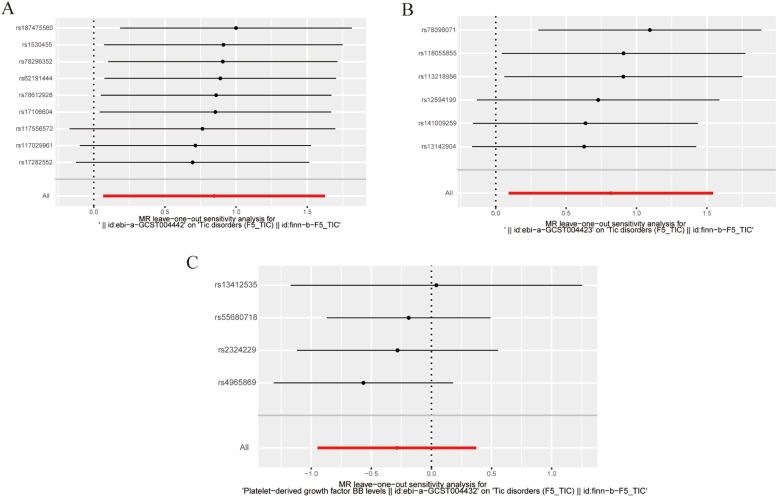
Leave-one-out results for IL-17, MIF, and PDGF-BB. (A) IL-17; (B) MIF; (C) PDGF-BB.

**Fig. 3:**
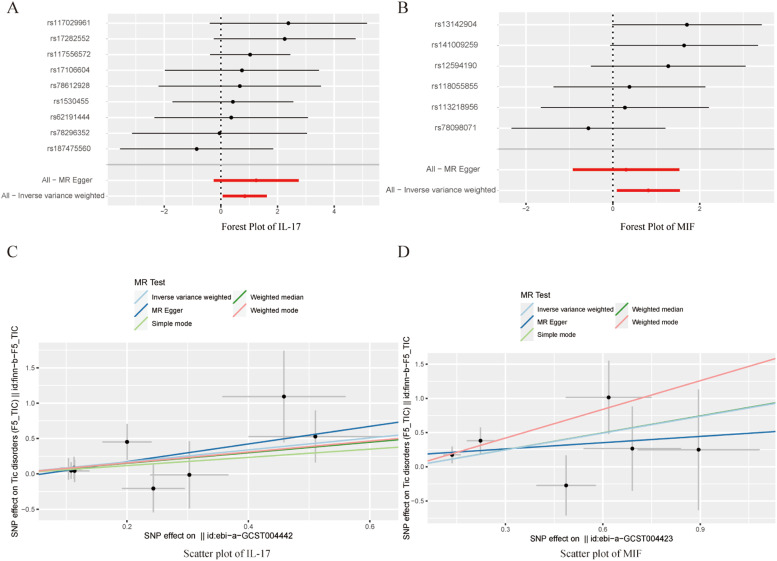
Forest plots and scatter plots for IL-17 and MIF.

**Fig. 4:**
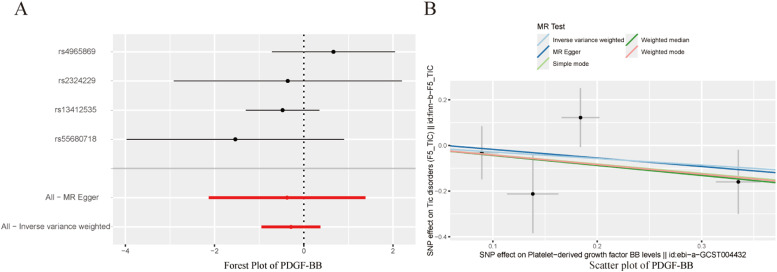
Forest plots and scatter plots for PDGF-BB.

**Fig. 5:**
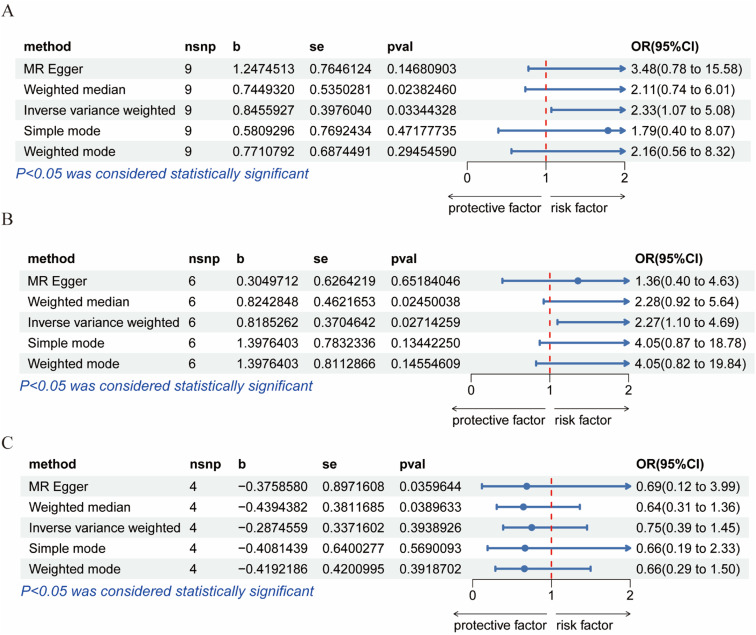
Risk Forest plot for IL-17, MIF, and PDGF-BB. (A) IL-17; (B) MIF; (C) PDGF-BB.

To avoid excessive bias, a series of sensitivity analyses were conducted to test the reliability of MR analysis and detect potential horizontal pleiotropy. As shown in Table 3, the intercept of MR-Egger indicates no horizontal pleiotropy for all causal effects (p > 0.05). Cochran's *Q* test and leave-one-out test suggested no significant heterogeneity. This indicates the robustness of the MR analysis results (see Fig. 2). In addition, MR power calculations show strong power (power > 80 %) in detecting significant causal effects. The leave-one-out plot in Fig. 2 demonstrates the stability of the MR analysis results. The forest plots in Figs. 3 (A‒B) and 4A display the individual and overall effects of SNPs related to IL-17, MIF, and PDGF-BB on TD. The scatter plots in Figs. 3 (C‒D) and 4B and the forest plot in Fig. 5 indicate that PDGF-BB may be a protective factor for TD, whereas IL-17 and MIF may be risk factors for TD (Fig. 4).

**Table 3:** Multifaceted analysis and heterogeneity examination of cytokine levels in TD patients.

	Genetic pleiotropy test			Heterogeneity test					
	MR Egger			MR Egger			IVW		
	Egger-intercept	SE	p	Q	Q_df	Q_pval	Q	Q_df	Q_pval
**IL-17**	-0.077	0.125	0.558	4.213	7.000	0.755	4.592	8.000	0.800
**MIF**	0.168	0.166	0.367	4.013	4.000	0.404	5.046	5.000	0.410
**PDGF-BB**	0.020	0.182	0.922	3.024	2.000	0.220	3.043	3.000	0.385

## Discussion

In this study, the authors used published GWAS data to infer causal relationships between 41 inflammatory factors in circulating blood and TD. The analysis results revealed that PDGF-BB can reduce the causal risk of TD, whereas excessive IL-17 and MIF can increase the risk of TD. In addition, the process of inferring causal relationships was not influenced by potential confounding factors, as evidenced by Cochran's *Q* and leave-one-out tests during the study, which did not find that any SNPs significantly affected the results. The MR-Egger method and MR-PRESSO test did not detect horizontal pleiotropy, and the MR statistical power values were all greater than 80 %, thereby increasing the reliability of the results. No causal relationship was found between other cell inflammatory factors and TD in the present study.

IL-17 plays a pivotal role in immune responses and inflammatory regulation.^14^ This proinflammatory cytokine is produced primarily by Th17 cells, a distinct subset of CD4+ T-cells that have garnered significant interest because of their involvement in the pathogenesis of various autoimmune and inflammatory diseases, such as rheumatoid arthritis, psoriasis, and multiple sclerosis.^15^^,^^16^ IL-17 exerts its biological effects by binding to its receptor, IL-17R, which is expressed in a variety of cell types, including fibroblasts, epithelial cells, endothelial cells, and macrophages.^17^ Following receptor binding, IL-17 activates multiple intracellular signaling pathways, particularly the NF-κB, MAPK, and PI3K-Akt pathways, leading to the induction of other proinflammatory cytokines (e.g., IL-6 and TNF-α), chemokines (e.g., CCL20 and CXCL1), and matrix metalloproteinases (e.g., MMP-1 and MMP-3), collectively amplifying the inflammatory response.^18^ Researchers have also shown interest in the association between IL-17 and neurofunctional disorders. The present findings suggest that elevated IL-17 levels may increase susceptibility to Tic Disorders (TDs). Cheng et al. reported significantly increased concentrations of IL-17 in the plasma of TD patients.^19^ Furthermore, Th17 cells and their IL-17 are actively implicated in various neurological disorders.^20^ IL-17 can directly affect brain cell development and indirectly negatively impact neural development by disrupting the Blood-Brain Barrier (BBB), causing neurovascular dysfunction, and through the gut-brain axis.^21^ Sreenivas et al. reported that in studies on neurodevelopmental disorders, the Compensatory Immune Regulatory System (CIRS) is activated, with increased IL-1 signaling, decreased levels of IL-1 receptor antagonists, and augmented levels of CCL2 and IL-17.^22^ Sallam DE et al. highlighted the involvement of IL-17 in regulating host defense against pathogens at barrier surfaces, tissue regeneration, and integration of the nervous, endocrine, and immune systems; in ASD patients, serum IL-17 levels were significantly greater than those in controls.^23^ Previous research has revealed immune dysregulation in TD patients, which may contribute to neuroinflammation. Microglia, through mediating neuroinflammation, modulating neuronal function, and participating in immune responses, might play crucial roles in the pathophysiology of TD.^24^^,^^25^ Zhou et al. demonstrated that IL-17 can induce microglial activation via the STAT3-iNOS pathway, promoting autoimmune reactions and impairing neuronal function, thereby exacerbating TD symptoms.^26^ Arenas et al. reported that in rats with hyperammonaemia and hepatic encephalopathy, increased IL-17 levels and membrane expression of IL-17 receptors in the cerebellum led to increased IL-17 receptor activation in microglia, triggering STAT3 and NF-kB activation and subsequently increasing IL-17 and TNFα levels.^27^ Waisman et al. proposed that targeting the functional inhibition of the IL-17 cytokine family could have beneficial effects on pathological conditions in the central nervous system.^28^ In the future, IL-17 may serve as a biomarker and therapeutic target for TD, although further investigation is needed.

MIF is a multifunctional cytokine that plays an important role in the immune response and inflammation processes. It is secreted by various cell types, including macrophages, T-cells, and endothelial cells.^29^ Its main function is to activate downstream signaling pathways, such as the MAPK and NF-κB signaling pathways, by binding to its receptor (e.g., CD74), thereby regulating the expression of inflammatory factors and the migration of immune cells.^30^ In the immune response, MIF can promote the activation and proliferation of T cells and macrophages, thereby enhancing the body's immune defense capabilities.^31^ Additionally, MIF can also inhibit the anti-inflammatory effects mediated by glucocorticoids, indicating its important role in maintaining the persistence and intensity of inflammatory responses.^32^ Studies have shown that MIF exacerbates tissue damage by promoting the secretion of chemokines and the expression of adhesion molecules, leading to the retention of inflammatory cells at the site of lesions.^33^ Furthermore, MIF can induce the expression of matrix metalloproteinases, promoting the degradation of the extracellular matrix and further exacerbating tissue inflammation and damage.^34^ MIF not only plays a role in the peripheral immune system but also participates in regulating neuroinflammation and neuronal function in the Central Nervous System (CNS).^35^ In the CNS, MIF is expressed mainly by neurons, astrocytes, and microglia. Its expression is significantly upregulated in neuroinflammatory and neurodegenerative diseases. It can penetrate the blood-brain barrier and directly affect the immune response of the CNS.^36^ By binding to its receptor CD74, MIF activates immune cells and glial cells, enhancing neuroinflammatory responses.^37^ You et al. reported that the plasma levels of MIF in the peripheral blood of TD patients were significantly greater than those in the control group.^38^ Although there is relatively little research on the role of MIF in the development of TD, it is interesting to note that the literature has documented elevated levels of MIF in the peripheral blood in various CNS diseases, such as depression, Parkinson's disease, Alzheimer's disease, multiple sclerosis, and stroke.^39^, ^40^, ^41^, ^42^, ^43^ Inácio et al. reported that MIF is part of the signaling network involved in brain plasticity and that elevated levels of MIF in neurons and/or astrocytes can inhibit the recovery of sensory-motor function after stroke. The downregulation of MIF may constitute a new therapeutic approach to promote the development and recovery of nerve fibers after stroke.^44^ Oikonomidi et al. demonstrated in a clinical trial that higher levels of MIF in cerebrospinal fluid are associated with accelerated decline in cognitive ability in MCI and mild dementia.^45^ A multilevel study of MIF in severe Depression (MDD) patients revealed that after three weeks of treatment, patients had significantly lower levels of MIF, but there was no strong evidence to support the utility of MIF as a biomarker for the diagnosis or monitoring of MDD.^46^ Park et al. reported that genetic depletion of MIF activity can prevent the loss of dopaminergic neurons and behavioral defects in a mouse model of Parkinson's disease, preventing neurodegenerative changes.^47^ In conclusion, the present study suggests that elevated MIF levels may increase the risk of TD development. Combined with the results of previous studies, the findings of this study suggest that MIF may be a promising therapeutic target for future TD treatment.

PDGF-BB plays crucial roles in the immune response and inflammatory processes. PDGF-BB not only has important functions in tissue repair and regeneration but also plays a critical role in the pathogenesis of various inflammatory and immune-related diseases.^48^ PDGF-BB exerts its biological effects through its receptors PDGFR-α and PDGFR-β, which are expressed mainly in smooth muscle cells, fibroblasts, and vascular endothelial cells.^49^ The binding of PDGF-BB activates multiple downstream signaling pathways, including the PI3K-Akt, MAPK, and STAT3 pathways, thereby promoting cell proliferation, migration, and survival.^50^ In the immune response, PDGF-BB significantly influences the function of immune cells. Studies have shown that PDGF-BB can regulate the polarization of macrophages, promoting their transition from the proinflammatory M1 phenotype to the anti-inflammatory M2 phenotype, thus assisting in inflammation resolution and tissue repair.^51^ During the acute inflammatory response, PDGF-BB accelerates tissue repair and regeneration by promoting the proliferation and migration of fibroblasts and smooth muscle cells.^52^ However, excessive PDGF-BB signaling may also lead to pathological fibrosis, increasing the risk of organ dysfunction.^53^ Recent research advances have revealed multiple roles of PDGF-BB in the CNS, including neuroprotection, neuroregeneration, and regulation of the neuroimmune response.^54^ In the central nervous system, PDGF-BB promotes the survival and function of neurons and glial cells. PDGF-BB exerts neuroprotective effects by activating downstream signaling pathways through its receptor PDGFR-β, such as the PI3K-Akt and MAPK pathways.^55^ The activation of these pathways helps to resist neuronal apoptosis and damage, particularly in neurodegenerative diseases such as Alzheimer's disease and Parkinson's disease.^56^ Furthermore, PDGF-BB promotes neuronal regeneration and axonal growth, accelerating the process of nerve repair following injury.^57^ In the neuroimmune response, PDGF-BB can suppress the excessive inflammatory response of activated microglia and astrocytes, thereby reducing damage from neuroinflammation. In the pathological process of multiple sclerosis, PDGF-BB slows the progression of the disease by regulating the integrity of the blood-brain barrier and promoting myelin regeneration.^58^ A cohort study by Narasimhalu et al. indicated that higher levels of PDGF-AB/BB were independently associated with a lower risk of recurrent vascular events, suggesting that PDGF-AB/BB may be a potential therapeutic target for stroke.^59^ Research by Smyth et al. demonstrated that PDGF-BB can promote pericyte proliferation and prevent apoptosis through ERK signaling and that supplementation with PDGF-BB signaling can stabilize the brain vascular system in Alzheimer's disease.^60^ PDGF-BB has been shown to have important neuroregenerative functions in various animal models of Parkinson's disease. Chen et al. reported that PDGF-BB can directly regulate the expression of tyrosine hydroxylase through the downstream Akt/ERK/CREB signaling pathway, playing a therapeutic role in Parkinson's disease.^61^ Although there is relatively little direct research on PDGF-BB in TD, its protective effects in other neurological diseases are evident. In conclusion, further cohort studies and clinical intervention studies are needed to determine whether PDGF-BB can serve as a marker or therapeutic target for TD on the basis of the impact of PDGF-BB on these findings.

The strength of this study lies in the use of MR analysis, based on large-scale GWAS data, ensuring the robustness of causal inference between inflammatory factors and TD. The study's high statistical power (> 80 %) and the lack of detected heterogeneity further support the reliability of these findings. Although the present study provides valuable insights, it is based on publicly available summary-level data and does not include individual-level data. Additionally, this analysis is limited to individuals of European ancestry, which may affect the generalizability of these results to other populations. Further cohort studies and clinical trials are needed to validate the findings in diverse populations.

The present study revealed that elevated levels of IL-17 and MIF in the circulating blood might serve as risk factors for TD patients, whereas high concentrations of PDGF-BB could be protective against TD onset. These findings offer a novel perspective on the relationship between circulating inflammatory cytokines and TD, thereby contributing to informed clinical decision-making. In terms of clinical application, identifying IL-17, MIF, and PDGF-BB as potential biomarkers for TD can guide the development of targeted prevention and treatment strategies. Elevated levels of IL-17 and MIF may provide information for risk assessment, while PDGF-BB may become a therapeutic target to alleviate TD onset. However, further in-depth research is needed to validate these results in the future.

## Authors’ contributions

CL conceived and wrote the manuscript and drew the drawings. GH, XC, XL, XL, WH, GL collected the references. AC supervised the research and revised the manuscript. All authors approved the submitted version.

## Funding

Supported by Shenzhen Hospital (Futian) of 10.13039/501100010618Guangzhou University of Chinese Medicine Research Project (Project No.: GZYSY2024013).

## Declaration of competing interest

The authors declare that the research was conducted in the absence of any commercial or financial relationships that could be construed as potential conflicts of interest.

## References

[1] Pringsheim T., Okun M.S., Müller-Vahl K. (2019). Practice guideline recommendations summary: treatment of tics in people with Tourette syndrome and chronic tic disorders. Neurology.

[2] Müller-Vahl K.R., Szejko N., Verdellen C. (2022). European clinical guidelines for Tourette syndrome and other tic disorders: summary statement. Eur Child Adolesc Psychiatry.

[3] Vermilion J., Mink J.W. (2023). Tic disorders. Pediatr Rev.

[4] Stiede J.T., Woods D.W. (2020). Pediatric prevention: Tic disorders. Pediatr Clin North Am.

[5] Singal A., Daulatabad D. (2017). Nail tic disorders: manifestations, pathogenesis and management. India J Dermatol Venereol Leprol.

[6] Sapozhnikov Y., Vermilion J. (2023). Co-occurring anxiety in youth with Tic disorders: a review. J Child Adolesc Psychopharmacol.

[7] Qi Y., Zheng Y., Li Z., Liu Z., Xiong L. (2019). Genetic studies of tic disorders and tourette syndrome. Method Mol Biol.

[8] Aman M., Coelho J.S., Lin B. (2022). Prevalence of pediatric acute-onset neuropsychiatric syndrome (PANS) in children and adolescents with eating disorders. J Eat Disord.

[9] Frick L., Pittenger C. (2016). Microglial dysregulation in OCD, tourette syndrome, and PANDAS. J Immunol Res.

[10] Jones H.F., Han V.X., Patel S. (2021). Maternal autoimmunity and inflammation are associated with childhood tics and obsessive-compulsive disorder: transcriptomic data show common enriched innate immune pathways. Brain Behav Immun.

[11] Larsson S.C., Butterworth A.S., Burgess S. (2023). Mendelian randomization for cardiovascular diseases: principles and applications. Eur Heart J.

[12] Birney E. (2022). Mendelian randomization. Cold Spring Harb Perspect Med.

[13] Burgess S., Thompson S.G. (2017). Interpreting findings from mendelian randomization using the MR‒Egger method. Eur J Epidemiol.

[14] Iwakura Y., Ishigame H., Saijo S., Nakae S. (2011). Functional specialization of interleukin-17 family members. Immunity.

[15] Sahu U., Biswas D., Prajapati V.K., Singh A.K., Samant M., Khare P. (2021). Interleukin-17-A multifaceted cytokine in viral infections. J Cell Physiol.

[16] Hadian Y., Bagood M.D., Dahle S.E., Sood A., Isseroff R.R. (2019). Interleukin-17: potential target for chronic wounds. Mediators Inflamm.

[17] Ramani K., Biswas P.S. (2019). Interleukin-17: friend or foe in organ fibrosis. Cytokine.

[18] Yao Y., Thomsen S.F. (2017). The role of interleukin-17 in the pathogenesis of hidradenitis suppurativa. Dermatol Online J.

[19] Cheng Y.H., Zheng Y., He F. (2012). Detection of autoantibodies and increased concentrations of interleukins in plasma from patients with Tourette's syndrome. J Mol Neurosci.

[20] Milovanovic J., Arsenijevic A., Stojanovic B. (2020). Interleukin-17 in chronic inflammatory neurological diseases. Front Immunol.

[21] Cipollini V., Anrather J., Orzi F., Iadecola C. (2019). Th17 and cognitive impairment: possible mechanisms of action. Front Neuroanat.

[22] Sreenivas N., Maes M., Padmanabha H. (2024). Comprehensive immunoprofiling of neurodevelopmental disorders suggests three distinct classes based on increased neurogenesis, Th-1 polarization or IL-1 signaling. Brain Behav Immun.

[23] Sallam D.E., Shaker Y.S., Mostafa G.A., El-Hossiny R.M., Taha S.I., Ahamed M.A.E.H. (2024). Evaluation of serum interleukin-17 A and interleukin-22 levels in pediatric patients with autism spectrum disorder: a pilot study. BMC Pediatr.

[24] Kawanokuchi J., Shimizu K., Nitta A. (2008). Production and functions of IL-17 in microglia. J Neuroimmunol.

[25] Frick L.R., Williams K., Pittenger C. (2013). Microglial dysregulation in psychiatric disease. Clin Dev Immunol.

[26] Zhou T., Liu Y., Yang Z. (2021). IL-17 signaling induces iNOS+ microglia activation in retinal vascular diseases. Glia.

[27] Arenas Y.M., López-Gramaje A., Montoliu C., Llansola M., Felipo V. (2024). Increased levels and activation of the IL-17 receptor in microglia contribute to enhanced neuroinflammation in cerebellum of hyperammonemic rats. Biol Res.

[28] Waisman A., Hauptmann J., Regen T. (2015). The role of IL-17 in CNS diseases. Acta Neuropathol.

[29] Sumaiya K., Langford D., Natarajaseenivasan K., Shanmughapriya S. (2022). Macrophage migration inhibitory factor (MIF): A multifaceted cytokine regulated by genetic and physiological strategies. Pharmacol Ther.

[30] Bilsborrow J.B., Doherty E., Tilstam P.V., Bucala R. (2019). Macrophage migration inhibitory factor (MIF) as a therapeutic target for rheumatoid arthritis and systemic lupus erythematosus. Expert Opin Ther Targets.

[31] Luo Y., Wang X., Shen J., Yao J. (2021). Macrophage migration inhibitory factor in the pathogenesis of leukemia (Review). Int J Oncol.

[32] Basile M.S., Battaglia G., Bruno V. (2020). The dichotomic role of macrophage migration inhibitory factor in neurodegeneration. Int J Mol Sci.

[33] Leyton-Jaimes M.F., Kahn J., Israelson A. (2018). Macrophage migration inhibitory factor: A multifaceted cytokine implicated in multiple neurological diseases. Exp Neurol.

[34] Osipyan A., Chen D., Dekker F.J. (2021). Epigenetic regulation in macrophage migration inhibitory factor (MIF)-mediated signaling in cancer and inflammation. Drug Discov Today.

[35] Grieb G. (2014). Macrophage migration inhibitory factor (MIF) and its receptors – interactions and suitability as biomarkers. Mini Rev Med Chem.

[36] Abidi J.H., Harris J., Deen N.S. (2020). Co-immunoprecipitation of macrophage migration inhibitory factor. Method Mol Biol.

[37] Swoboda C., Deloch L., von Zimmermann C. (2022). Macrophage migration inhibitory factor in major depressive disorder: a multilevel pilot study. Int J Mol Sci.

[38] You H.Z., Zhang J., Du Y. (2023). Association of elevated plasma CCL5 levels with high risk for tic disorders in children. Front Pediatr.

[39] Petralia M.C., Mazzon E., Fagone P. (2020). Pathogenic contribution of the Macrophage migration inhibitory factor family to major depressive disorder and emerging tailored therapeutic approaches. J Affect Disord.

[40] Li S., Nie K., Zhang Q. (2019). Macrophage migration inhibitory factor mediates neuroprotective effects by regulating inflammation, apoptosis and autophagy in Parkinson's Disease. Neuroscience.

[41] Petralia M.C., Battaglia G., Bruno V. (2020). The role of macrophage migration inhibitory Factor in Alzheimer's Disease: conventionally pathogenetic or unconventionally protective?. Molecules.

[42] Ladakis D.C., Reyes-Mantilla M.I., Gadani S.P. (2024). Serum macrophage migration inhibitory factor levels predict brain atrophy in people with primary progressive multiple sclerosis. Mult Scler.

[43] Xuan W., Xie W., Li F. (2023). Dualistic roles and mechanistic insights of macrophage migration inhibitory factor in brain injury and neurodegenerative diseases. J Cereb Blood Flow Metab.

[44] Inácio A.R., Ruscher K., Wieloch T. (2011). Enriched environment downregulates macrophage migration inhibitory factor and increases parvalbumin in the brain following experimental stroke. Neurobiol Dis.

[45] Oikonomidi A., Tautvydaitė D., Gholamrezaee M.M., Henry H., Bacher M., Popp J. (2017). Macrophage migration inhibitory factor is associated with biomarkers of Alzheimer's Disease pathology and predicts cognitive decline in mild cognitive impairment and mild dementia. J Alzheimers Dis.

[46] Swoboda C., Deloch L., von Zimmermann C. (2022). Macrophage migration inhibitory factor in major depressive disorder: a multilevel pilot study. Int J Mol Sci.

[47] Park H., Kam T.-I., Peng H. (2022). PAAN/MIF nuclease inhibition prevents neurodegeneration in Parkinson's disease. Cell.

[48] Wang C., Liu Y., He D. (2019). Diverse effects of platelet-derived growth factor-BB on cell signaling pathways. Cytokine.

[49] Komatsu K., Ideno H., Shibata T., Nakashima K., Nifuji A. (2022). Platelet-derived growth factor-BB regenerates functional periodontal ligament in the tooth replantation. Sci Rep.

[50] Chen H., Teng Y., Chen X. (2021). Platelet-derived growth factor (PDGF)-BB protects dopaminergic neurons via activation of Akt/ERK/CREB pathways to upregulate tyrosine hydroxylase. CNS Neurosci Ther.

[51] Ke Y., Bi X., Yang N. (2022). Serum platelet-derived growth factor-BB levels as a potential biomarker in assessing the metabolic activity of lesions in alveolar echinococcosis patients. Acta Trop.

[52] Idemoto K., Ishima T., Niitsu T. (2021). Platelet-derived growth factor BB: A potential diagnostic blood biomarker for differentiating bipolar disorder from major depressive disorder. J Psychiatr Res.

[53] Ren F., Fang Q., Xi H., Feng T., Wang L., Hu J. (2020). Platelet-derived growth factor-BB and epidermal growth factor promote dairy goat spermatogonial stem cells proliferation via Ras/ERK1/2 signaling pathway. Theriogenology.

[54] Okura Y., Imao T., Murashima S. (2019). Interaction of nerve growth factor β with adiponectin and SPARC oppositely modulates its biological activity. Int J Mol Sci.

[55] Changlong Z., Guangwei Z., Xuenong H., Xiaohui X., Xiaochuan S., Yanfeng X. (2016). The role of platelet-derived growth factor receptor in early brain injury following subarachnoid hemorrhage. J Stroke Cerebrovasc Dis.

[56] Lobsiger C.S., Schweitzer B., Taylor V., Suter U. (2000). Platelet-derived growth factor-BB supports the survival of cultured rat Schwann cell precursors in synergy with neurotrophin-3. Glia.

[57] Forsberg-Nilsson K., Behar T.N., Afrakhte M., Barker J.L., McKay R.D. (1998). Platelet-derived growth factor induces chemotaxis of neuroepithelial stem cells. J Neurosci Res.

[58] Oya T., Zhao Y.-L., Takagawa K. (2002). Platelet-derived growth factor-b expression induced after rat peripheral nerve injuries. Glia.

[59] Narasimhalu K., Ma L., De Silva D.A., Wong M.C., Chang H.M., Chen C. (2015). Elevated platelet-derived growth factor AB/BB is associated with a lower risk of recurrent vascular events in stroke patients. Int J Stroke.

[60] Smyth L.C.D., Highet B., Jansson D. (2022). Characterization of PDGF-BB:pdgfrβ signaling pathways in human brain pericytes: evidence of disruption in Alzheimer's disease. Commun Biol.

[61] Chen H., Teng Y., Chen X. (2021). Platelet-derived growth factor (PDGF)-BB protects dopaminergic neurons via activation of Akt/ERK/CREB pathways to upregulate tyrosine hydroxylase. CNS Neurosci Ther.

